# Added Value of QuantiFERON TB-Gold in-Tube for Detecting Latent Tuberculosis Infection among Persons Living with HIV/AIDS

**DOI:** 10.1155/2014/294963

**Published:** 2014-05-29

**Authors:** Josiane Maria Oliveira Souza, Maria do Socorro Nantua Evangelista, Anete Trajman

**Affiliations:** ^1^University of Brasilia (UnB), Campus Universitário Darcy Ribeiro, 70910-900 Brasília, DF, Brazil; ^2^SHA, Conjunto 5 chácara 47, Quadra D, Arniqueiras, 71995-297 Taguatinga, DF, Brazil; ^3^Catholic University of Brasilia (UCB), Campus I, QS 07 Lote 01 EPCT, Águas Claras, 71966-700 Taguatinga, DF, Brazil; ^4^Federal University of Rio de Janeiro (UFRJ), Avenida Brigadeiro Trompowsky s/n°, Ilha do Fundão Prédio do Hospital Universitário Clementino Fraga Filho, 11° andar Bloco F, Sala 4, 21941-590 Rio de Janeiro, RJ, Brazil; ^5^Montreal Chest Institute, McGill University, 3650 St. Urbain Street, Montreal, QC, Canada H2X 2P4

## Abstract

*Objective*. To evaluate the added value of QuantiFERON TB-Gold in-Tube (QTF-GIT) over the tuberculin skin testing (TST) for detecting latent tuberculosis (TB) infection (LTBI) among patients with AIDS in a city with a low TB incidence rate (11.1/100,000 inhabitants) and universal BCG coverage. *Methods*. Three hundred consecutive patients with AIDS in eight outpatient sexually transmitted disease public clinics in Brasilia were submitted to QFT-IT and TST between May 2011 and March 2013. A positive result of either test was considered to be LTBI. *Results*. Median CD4-cell count was 477.5 cells/mm^3^; 295 (98.3%) were using antiretroviral therapy. Eighteen patients (6%, 95% CI: 3.6%–9.3%) had LTBI, of whom 4 (1.3%, 95% CI: 0.04%–2.63%) had only a positive TST, 8 (2.7%, 95% CI: 0.8%–4.5%) had only a QFT-GIT positive test, and 6 (2%, 95% CI: 0.4%–3.6%) had positive results for both tests. This represents an 81.8% relative increase in LTBI detection when QFT-GIT is added to TST. The concordance between both tests was 96% (*k* = 0.48). *Conclusions*. The QFT-GIT alone was more effective to detect LTBI than TST alone and had an 81% added value as an add-on sequential test in this population with mild immunosuppression. The cost-effectiveness of these strategies remains to be evaluated.

## 1. Introduction

Persons living with HIV/AIDS (PLWHA) have a 20- to 37-fold increased risk for developing active tuberculosis (TB) [1], which is a leading cause of death among them [2, 3]. Treatment of latent tuberculosis infection (LTBI) can significantly reduce the risk of progression for active TB and of death among PLWHA [1, 3–5]. Thus, detection and treatment of latent TB infection (LTBI) is a priority in this population, and periodic testing is recommended in many guidelines [6–9].

Tuberculin skin testing (TST) has been used to detect LTBI for over a century. However, there are many limitations to its programmatic, public health use: reduced sensitivity in PLWHA, reduced specificity in BCG vaccinated populations (in particular when repeated tests are needed), need for two visits for tuberculin injection and induration reading, and need for adequate training of healthcare workers (HCW) [10, 11]. Interferon-gamma release assays (IGRAs) have the advantage of one unique visit and are less operator-dependent [11].

All tests for LTBI detection have lower sensitivity in PLWHA [11, 12]. Although it is controversial, in most studies comparing IGRAs in PLWHA, the T-SPOT.TB (Oxford Immunotec, Abingdon, UK), an Elispot technique-based IGRA, had a slightly better accuracy [11, 13] than QuantiFERON TB-Gold in-Tube (QFT-GIT, Cellestis, Carnegie, Australia), an ELISA-based IGRA which has been adopted by many developed countries for LTBI detection [6, 14–17] in this population. Nevertheless, IGRAs have not been considered cost-effective in immunocompetent adults from the National Public Health System in Brazil (SUS) [18].

The objective of the present study was to evaluate the added value of QFT-GIT over the TST for detecting LTBI among PLWHA in Brasilia, the capital of Brazil, a city with a low TB incidence rate (11.1/100.000 inhabitants) and universal coverage by BCG. We also explored the factors associated with a positive QFT-GIT and with discordant QFT-GIT/TST results.

## 2. Methods

A cross-sectional study was conducted in eight outpatient sexually transmitted disease public clinics between May 2011 and March 2013. Patients diagnosed with AIDS according to Rio de Janeiro/Caracas or adapted CDC criteria [19] living in Brasilia, Federal District, were eligible. Those over 17 years who were not submitted to TST in the previous five weeks were consecutively invited to participate and included if they signed an informed consent. Patients with history of other immunosuppression conditions were excluded: severe AIDS-related opportunistic infections, acute viral infections, those submitted to any vaccination in the previous two months, and those using immunosuppressive drugs. Patients with present or past active TB and those with a history of a previous positive TST were also excluded. The sample size was calculated to be 300 to have an 80% power, considering a 5% type I error to detect an association of a positive test with six variables (patients' characteristics).

After signing the informed consent, participants answered a questionnaire about sociodemographic, clinical, and epidemiological information. CD4-cell count and antiretroviral therapy data (ART) were extracted from medical records. Participants were then submitted to TST using 0.1 mL of PPD-RT 23 (corresponding to 2 units of tuberculin). Injection and reading of induration 72 to 96 hours after injection were performed by a trained HCW. QFT-GIT was performed according to the manufacturer's instruction [20].

Patients were considered to have LTBI if TST induration was ≥5 mm or if the difference between interferon response to TB antigens and negative control was ≥0.35 UI/mL and interferon response to TB antigens was ≥ 25% compared to the negative control response. QFT-GIT was considered to be indeterminate if the interferon response to the negative control was ≥8 UI/mL or <0.5 UI/mL compared to the positive control. Although the Brazilian guidelines [21] recommend treating only those with a positive TST, all participants considered to have LTBI, regardless of the test, were forwarded to preventive isoniazid therapy [11, 16].

The number of detected LTBI cases by any test and the number of additional LTBI cases detected by a positive QFT-GIT test as an add-on test as well as the number necessary to test (NTT) and their exact 95% confidence intervals (CI) were calculated. Concordance of test results was compared using kappa statistics. The association of independent variables and a positive result was estimated by the adjusted odds ratio (OR) in a multivariate model using logistic regression using the SPSS package, version 20 (Chicago, IL).

The study was approved by the Institutional Review Board of the Federal District Health Secretariat (Document 0019/2011).

## 3. Results

The characteristics of the 300 participants according to their LTBI status are displayed in Table 1. Their median age was 40 (interquartile range: IQR = 32–46) years; median CD4 cell count was 477.5 (95% CI: 450.5–504.5) cells/mm³; 295 (98.3%) were using ART. Eighteen (6.0%, 95% CI: 3.6%–9.3%) had LTBI, of whom 10 (55.6%, 95% CI: 30.8%–78.5%) were detected with TST. Out of those, 6 (60%, 95% CI: 26.2%–87.8%) and an additional 8 had a positive QFT-GIT. In other words, 3.3% (95% CI: 1.3%–5.7%) had a positive TST, out of whom 1.3% could only be detected by a positive TST, while 2.7%, nearly half of those with LTBI, could only be detected with the QFT-GIT test, representing an 81.8% relative increase in LTBI detection when added to TST. One QFT-GIT had an indeterminate result. Although income was not significantly associated with LTBI (using a cut-off of 1 minimum wage, OR = 2.1; 95% CI: 0.8–5.9; *P* = 0.16), lower schooling was as follows: all patients with LTBI had under 9 years of schooling (OR = 47.6; 95% CI: 6.3–361.6; *P* < 10^−8^).

The NNT to detect one LTBI case was 30 using TST as a single test and 21.4 using QFT-GIT as a single test; the NNT with QFT as an add-on test to detect one additional case was 36.2. The concordance between both tests was 96% (*k* = 0.48), mainly due to agreement on negative cases (Table 2). There was no significant association of the independent variables with a positive test result (Tables 3 and 4) or with discordant results (Table 5).

## 4. Discussion

In the present study, the prevalence of LTBI in PLWHA was low (6%) [22–25] considering the population age. Although these data are consistent with other tuberculin surveys in Brazil [26, 27] and other countries [22, 23, 28], this can be an underestimate, since sensitivity of both TST and QFT-GIT is suboptimal in PLWHA [11, 12]. However, among those with lower educational background, prevalence was much higher (22.5%), which confirms previous findings that TB infection is associated with socioeconomic class [29].

More importantly, QFT-GIT alone was more effective to detect LTBI than TST, assuming that any test is a marker of LTBI. This is important because despite absence of substantial evidence of the benefit of isoniazid preventive therapy in QFT-GIT positive patients, recent studies have shown a high risk of active TB in these patients [2, 7, 29–31] and thus treatment of PLWHA with discordant tests has been recommended [15, 16] considering the high efficacy of isoniazid preventive therapy [3, 5, 32] to prevent disease and death among these patients.

The added value of QFT-GIT in PLWHA has been explored in other countries with low burden and incidence rate of TB (Spain and USA), with variable findings. More modest effects of QFT in the absolute (less than 1.4%) and relative (less than 20%) increase in LTBI detection have been reported [22, 24, 28]. On the other hand, another study in Spain showed a similar result to ours: an absolute increase in 2.9% and relative increase in 47.8% (from 6.7% to 9.6%) [23]. Another research carried out in Germany showed a higher increase in the QTF-GIT outcomes when compared to the TST, this time of 7.2% [25].

The best approach for LTBI screening in PLWHA remains to be defined and will likely be variable in different scenarios and populations, depending on TB incidence rates and stage of the disease. In our study, QFT-GIT as an add-on sequential test to TST doubled the detection of LTBI. However, due to the large difference in costs of both tests [11, 33], a cost-effectiveness analysis considering both strategies is necessary. A cost-effectiveness study of QFT-GIT as an add-on test to confirm LTBI in Brazil has shown that this strategy was dominated (less effective and more costly). However, in this analysis, the base cases were immunocompetent individuals [18]. Moreover, no analysis was done to evaluate the added value for detecting more cases, since isoniazid preventive therapy is indicated in the country only for TST positive patients at present [21]. WHO does not recommend the use of IGRA as a replacement test for TST for PLWHA in poor low- and medium-income countries [33]. In case the Brazilian National TB Program considers following the CDC recommendations [8], cost-effectiveness analyses considering PLWHA and the added value of QFT-GIT to detect new LTBI cases will be necessary.

The study has a few limitations. TST is a reader-dependent technique, which always raises concerns about its accuracy. However, all HCW in the present study were previously trained for injection of tuberculin and reading of induration. The low prevalence of positive results has limited the capacity to evaluate risk factors associated to positive results and to discordance associated factors. The transversal study design also hindered prospectively evaluating the TB incidence. These possible limitations are outweighed by its strengths: being a pragmatic study with routine data collection in the public health sector and responsible for assistance of over 1.5% of the Brazilian population with AIDS [34]. Although we should not generalize the present results to other populations, we believe that the present findings show a definite value of QFT-GIT in PLWHA in a medium-income country in a low TB prevalence scenario.

## 5. Conclusions

The QFT-GIT alone was more sensitive to detecting LTBI than TST and doubled the detection of LTBI when used as an add-on sequential test in this population with mild immunosuppression, in a region with low incidence rates of TB and universal coverage by BCG. However, due to the large difference in costs of both tests, a cost-effectiveness analysis considering both strategies is necessary among PLWHA.

## Figures and Tables

**Table 1:** Characteristics of 300 participants with AIDS according to the presence of LTBI detected by TST or QFT-GIT in Brasilia, Federal District, Brazil.

Characteristics	With LTBI (*n* = 18) *n* (%)	Without LTBI (*n* = 282) *n* (%)
Sex		
Male	15 (7.0)	200 (93.0)
Female	3 (3.5)	82 (96.5)
Schooling (years)		
<9 years	18 (18.4)	80 (81.6)
9–12 years	—	105 (100)
>12 years	—	97 (100)
Marital status		
Single	12 (7.2)	154 (92.8)
Married	2 (3.8)	51 (96.2)
Divorced	2 (8.3)	22 (91.7)
Widower	—	11 (100)
Living together	2 (4.3)	44 (95.7)
Monthly income*		
None	2 (8.7)	21 (91.3)
<1 minimum wage	5 (10.0)	45 (90.0)
1–5 minimum wages	7 (4.4)	152 (95.6)
>5 minimum wage	4 (5.9)	64 (94.1)
Alcohol intake		
Yes	11 (6.9)	148 (93.1)
No	7 (5.0)	134 (95.0)
Smoking habit		
Yes (ever)	1 (3.8)	25 (96.2)
No	17 (6.2)	257 (93.8)
BCG vaccination		
Yes	15 (6.6)	213 (93.4)
No	3 (4.2)	69 (95.8)
Doses of BCG^†^		
One	14 (6.7)	196 (93.3)
Two	1 (5.6)	17 (94.4)
History of contact with index case		
No	16 (6.2)	243 (93.8)
Yes	1 (2.9)	34 (97.1)
No information	1 (16.7)	5 (83.3)
CD4 count		
≥200 cells/mm³	17 (6.3)	253 (93.7)
<200 cells/mm³	1 (3.3)	29 (96.7)

Legend: LTBI: latent tuberculosis infection; BCG: Bacillus Calmette-Guérin.

*Minimum wage = 615.00 Brazilian reais, equivalent to 349.40 American dollars at the time of data collection.

^†^Percentage calculated over those vaccinated with BCG.

**Table 2:** Agreement between TST and QFT test results in 300 patients with AIDS in Brasilia, Federal District, Brazil.

		TST		
		≥5 mm	<5 mm	Total
QFT-GIT	Positive	6	8	14
	Negative	4	281	285
	Indeterminate	0	1	1
Total		**10**	**290**	**300**

NOTE: Kappa = 0.48; *P*-value <0.001.

Legend: TST: tuberculin skin testing; QTF-GIT: QuantiFERON TB-Gold in-Tube.

**Table 3:** TST results according to characteristics of 300 patients with AIDS in Brasilia, Federal District, Brazil.

Characteristics	TST positive *n* (%)	TST negative *n* (%)	Nonadjusted OR (95% CI)	Adjusted OR (95% CI)
Sex				
Male	9 (4.2)	206 (95.8)	3.67 (0.46–29.42)	4.27 (0.40–45.58)
Female	1 (1.2)	84 (98.8)	1.0 (reference)	1.0 (reference)
Schooling (years)				
<9 years	2 (3.4)	56 (96.6)	1.04 (0.22–5.05)	1.26 (0.20–7.95)
≥9 years	8 (3.3)	234 (96.7)	1.0 (reference)	1.0 (reference)
Single				
Yes	7 (4.2)	159 (95.8)	1.92 (0.49–7.58)	1.37 (0.30–6.33)
No	3 (2.2)	131 (97.8)	1.0 (reference)	1.0 (reference)
Monthly income*				
None	2 (8.7)	21 (91.3)	2.06 (0.32–13.20)	2.37 (0.28–20.02)
<1 minimum wage	2 (4.0)	48 (96.0)	0.90 (0.14–5.61)	1.88 (0.21–16.83)
1–5 minimum wages	3 (1.9)	156 (98.1)	0.42 (0.08–2.12)	0.59 (0.11–3.29)
>5 minimum wages	3 (4.4)	65 (95.6)	1.0 (reference)	1.0 (reference)
Alcohol intake				
Yes	8 (5.0)	151 (95.0)	3.68 (0.77–17.64)	2.52 (0.49–12.82)
No	2 (1.4)	139 (98.6)	1.0 (reference)	1.0 (reference)
Smoking habit				
Yes (ever)	1 (2.9)	34 (97.1)	0.84 (0.10–6.67)	0.72 (0.08–6.25)
No	9 (3.4)	256 (96.6)	1.0 (reference)	1.0 (reference)
BCG vaccination				
Yes	8 (3.5)	220 (96.5)	1.27 (0.26–6.13)	1.13 (0.20–6.35)
No	2 (2.8)	70 (97.2)	1.0 (reference)	1.0 (reference)
History of contact with index case				
Yes	1 (2.9)	34 (97.1)	0.92 (0.11–7.61)	1.21 (0.13–11.16)
No	8 (3.1)	251 (96.9)	1.0 (reference)	1.0 (reference)
No information	1 (16.7)	5 (83.3)	—	—
CD4 count,^§^				
<200 cells/mm³	—	30 (100)	0.76 (0.10–6.25)	—
≥200 cells/mm³	10 (3.7)	260 (96.3)	1.0 (reference)	—
Doses of BCG,^†,§^				
One	8 (3.8)	202 (96.2)	0.00	—
Two	—	18 (100)	1.0 (reference)	—

Legend: LTBI: latent tuberculosis infection; BCG: Bacillus Calmette-Guérin; TST: tuberculin skin testing.

*Minimum wage = 615.00 Brazilian reais, equivalent to 349.40 American dollars at the time of data collection.

^†^Percentage calculated over those vaccinated with BCG.

^§^One unit added to all cells to allow OR calculation.

**Table 4:** QFT-GIT results according to characteristics of 300 patients with AIDS in Brasilia, Federal District, Brazil.

Characteristics	QTF-GIT positive *n* (%)	QTF-GIT negative *n* (%)	Nonadjusted OR (95% CI)	Adjusted OR (95% CI)
Sex				
Male	12 (5.6)	202 (94.4)	2.46 (0.54–11.26)	4.70 (0.86–25.59)
Female	2 (2.4)	83 (97.6)	1.0 (reference)	1.0 (reference)
Schooling (years)				
<9 years	5 (8.6)	53 (91.4)	2.43 (0.78–7.55)	2.41 (0.65–8.87)
≥9 years	9 (3.7)	232 (96.3)	1.0 (reference)	1.0 (reference)
Single				
Yes	9 (5.5)	156 (94.5)	1.49 (0.49–4.55)	1.19 (0.35–4.03)
No	5 (3.7)	129 (96.3)	1.0 (reference)	1.0 (reference)
Monthly income*				
None	2 (8.7)	21 (91.3)	6.38 (0.55–73.94)	6.55 (0.50–86.21)
<1 minimum wage	4 (8.0)	46 (92.0)	5.83 (0.63–53.81)	7.40 (0.66–82.21)
1–5 minimum wages	7 (4.4)	151 (95.6)	3.11 (0.37–25.75)	3.09 (0.35–26.91)
>5 minimum wages	1 (1.5)	67 (98.5)	1.0 (reference)	1.0 (reference)
Alcohol intake				
Yes	8 (5.1)	150 (94.9)	1.20 (0.41–3.55)	1.09 (0.34–3.49)
No	6 (4.3)	135 (95.7)	1.0 (reference)	1.0 (reference)
Smoking habit				
Yes (ever)	1 (2.9)	34 (97.1)	0.57 (0.07–4.54)	0.44 (0.05–3.70)
No	13 (4.9)	251 (95.1)	1.0 (reference)	1.0 (reference)
BCG vaccination				
Yes	12 (5.3)	215 (94.7)	1.95 (0.43–8.94)	2.09 (0.16–28.05)
No	2 (2.8)	70 (97.2)	1.0 (reference)	1.0 (reference)
Doses of BCG^†^				
Two	1 (5.6)	17 (94.4)	0.94 (0.11–7.76)	0.81 (0.09–7.47)
One	11 (5.3)	198 (94.7)	1.0 (reference)	1.0 (reference)
CD4 count				
<200 cells/mm³	1 (3.3)	29 (96.7)	0.68 (0.08–5.26)	0.58 (0.07–5.00)
≥200 cells/mm³	13 (4.8)	256 (95.2)	1.0 (reference)	1.0 (reference)
History of contact with index case^§^				
Yes	—	35 (100)	0.49 (0.06–3.82)	—
No	13 (5.0)	245 (95.0)	1.0 (reference)	—
No information	1 (16.7)	5 (83.3)	—	—

Legend: LTBI: latent tuberculosis infection; BCG: Bacillus Calmette-Guérin; QTF-GIT: QuantiFERON TB-Gold in-Tube.

*Minimum wage = 615.00 Brazilian reais, equivalent to 349.40 American dollars at the time of data collection.

^†^Percentage calculated over those vaccinated with BCG.

^§^One unit added to all cells to allow OR calculation.

**Table 5:** Discordant and concordant results of QTF-GIT and TST according to characteristics of 300 patients with AIDS in Brasilia, Federal District, Brazil.

Characteristics	Discordant result *n* (%)	Concordant result *n* (%)	Nonadjusted OR (95% CI)	Adjusted OR (95% CI)
Sex				
Male	9 (4.2)	206 (95.8)	0.83 (0.22–3.16)	0.59 (0.12–2.86)
Female	3 (3.5)	82 (96.5)	1.0 (reference)	1.0 (reference)
Schooling (years)				
<9 years	5 (8.6)	53 (91.4)	1.0 (reference)	1.0 (reference)
≥9 years	7 (2.9)	234 (97.1)	3.15 (0.96–10.32)	6.31 (1.28–31.22)
Single				
Yes	8 (4.8)	157 (95.2)	1.0 (reference)	1.0 (reference)
No	4 (3.0)	130 (97.0)	1.66 (0.49–5.62)	1.57 (0.41–5.98)
Monthly income^∗ §^				
None	—	23 (100)	—	—
<1 minimum wage	4 (8.0)	46 (92.0)	0.72 (0.17–3.02)	1.73 (0.25–12.22)
1–5 minimum wages	4 (2.5)	155 (97.5)	2.41 (0.58–9.92)	3.85 (0.78–18.96)
>5 minimum wages	4 (5.9)	64 (94.1)	1.0 (reference)	1.0 (reference)
Alcohol intake				
Yes	6 (3.8)	152 (96.2)	1.13 (0.35–3.57)	1.15 (0.34–3.92)
No	6 (4.3)	135 (95.7)	1.0 (reference)	1.0 (reference)
BCG vaccination				
Yes	10 (4.4)	218 (95.6)	1.0 (reference)	1.0 (reference)
No	2 (2.8)	70 (97.2)	1.61 (0.34–7.54)	5.47 (0.35–84.32)
Doses of BCG^†^				
Two	1 (5.6)	17 (94.4)	1.31 (0.16–10.94)	2.48 (0.24–25.64)
One	9 (4.3)	201 (95.7)	1.0 (reference)	1.0 (reference)
CD4 count				
<200 cells/mm³	1 (3.3)	29 (96.7)	1.24 (0.15–9.92)	1.27 (0.13–12.22)
≥200 cells/mm³	11 (4.1)	258 (95.9)	1.0 (reference)	1.0 (reference)
Smoking habit^§^				
Yes (ever)	—	35 (100)	1.85 (0.23–14.57)	—
No	12 (4.5)	253 (95.5)	1.0 (reference)	—
History of contact with index case^§^				
Yes	1 (2.9)	34 (97.1)	0.00	—
No	11 (4.3)	248 (95.7)	1.0 (reference)	—
No information	—	6 (100)	—	—

Legend: LTBI: latent tuberculosis infection; BCG: Bacillus Calmette-Guérin; TST: tuberculin skin testing; QTF-GIT: QuantiFERON TB-Gold in-Tube.

*Minimum wage = 615.00 Brazilian reais, equivalent to 349.40 American dollars at the time of data collection.

^†^Percentage calculated over those vaccinated with BCG.

^§^One unit added to all cells to allow OR calculation.
